# CYLD/HDAC6 signaling regulates the interplay between epithelial-mesenchymal transition and ciliary homeostasis during pulmonary fibrosis

**DOI:** 10.1038/s41419-024-06972-4

**Published:** 2024-08-09

**Authors:** Hua Ni, Miao Chen, Dan Dong, Yunqiang Zhou, Yu Cao, Ruixin Ge, Xiangrui Luo, Yutao Wang, Xifeng Dong, Jun Zhou, Dengwen Li, Songbo Xie, Min Liu

**Affiliations:** 1 State Key Laboratory of Medicinal Chemical Biology, Haihe Laboratory of Cell Ecosystem, College of Life Sciences, Nankai University, Tianjin, China; 2 Key Laboratory of Biological Resources and Ecology of Pamirs Plateau in Xinjiang Uygur Autonomous Region, College of Life and Geographic Sciences, Kashi University, Kashi, China; 3 School of Life Sciences and Medicine, Shandong University of Technology, Zibo, China; 4 Center for Cell Structure and Function, Collaborative Innovation Center of Cell Biology in Universities of Shandong, College of Life Sciences, Shandong Normal University, Jinan, China; 5 Department of Hematology, Tianjin Key Laboratory of Bone Marrow Failure and Malignant Hemopoietic Clone Control, Tianjin Institute of Hematology, Tianjin Medical University General Hospital, Tianjin, China; 6 Department of Ophthalmology, Tianjin Medical University General Hospital, Ministry of Education International Joint Laboratory of Ocular Diseases, Tianjin Key Laboratory of Ocular Trauma, Tianjin Institute of Eye Health and Eye Diseases, China-UK “Belt and Road” Ophthalmology Joint Laboratory, Haihe Laboratory of Cell Ecosystem, Tianjin Medical University, Tianjin, 300052 China; 7 Laboratory of Tissue Homeostasis, Haihe Laboratory of Cell Ecosystem, Tianjin, China

**Keywords:** Transdifferentiation, Respiratory tract diseases

## Abstract

The primary cilium behaves as a platform for sensing and integrating extracellular cues to control a plethora of cellular activities. However, the functional interaction of this sensory organelle with epithelial-mesenchymal transition (EMT) during pulmonary fibrosis remains unclear. Here, we reveal a critical role for cylindromatosis **(**CYLD) in reciprocally linking the EMT program and ciliary homeostasis during pulmonary fibrosis. A close correlation between the EMT program and primary cilia is observed in bleomycin-induced pulmonary fibrosis as well as TGF-β-induced EMT model. Mechanistic study reveals that downregulation of CYLD underlies the crosstalk between EMT and ciliary homeostasis by inactivating histone deacetylase 6 (HDAC6) during pulmonary fibrosis. Moreover, manipulation of primary cilia is an effective means to modulate the EMT program. Collectively, these results identify a pivotal role for the CYLD/HDAC6 signaling in regulating the reciprocal interplay between the EMT program and ciliary homeostasis during pulmonary fibrosis.

## Introduction

Pulmonary fibrosis is a multifactorial disease characterized by proliferation of fibroblasts and deposits of a large amount of extracellular matrix, accompanied by inflammatory damage and lung tissue structure destruction [1, 2]. It threatens the health of approximately 1.7 million of people worldwide. The mortality rate of pulmonary fibrosis is very high, with a reported median survival of 2–3 years after diagnosis, while the incidence rate is increasing year by year [3]. Several cytokines, including transforming growth factor β1 (TGF-β1), platelet-derived growth factor (PDGF), vascular endothelial growth factor (VEGF), and fibroblast growth factor (FGF), are critically involved in the pathophysiological process of pulmonary fibrosis [4]. Although drugs targeting these signaling pathways, such as pirfenidone (an antifibrotic agent that reduces the TGF-β level) and nintedanib (a VEGFR/FGFR/PDGFR inhibitor), are clinically approved by the US Food and Drug Administration for the treatment of pulmonary fibrosis, they can only slow down the decline rate of pulmonary function. They are not effective for all patients and may have unsatisfactory side effects in some cases [5, 6]. Therefore, it is necessary to further elucidate the pathophysiological mechanisms of pulmonary fibrosis and discover new treatment strategies.

Progressive scarring resulting from abnormal repair of normal lung alveolar tissue after damage is the culprit of pulmonary fibrosis. During this process, epithelial-to-mesenchymal transition (EMT) is an important step that can be activated by several intracellular signaling pathways, with the TGF-β signaling cascade playing a central role [7]. TGF-β binds to type I and II receptor complexes, leading to the activation of SMAD complexes, which subsequently enter the nucleus to regulate the transcription of mesenchymal genes [8]. In addition to this canonical signaling pathway, TGF-β also activates a plethora of noncanonical pathways involving PI3K-AKT, p38 MAPK, and ERK [9]. Ultimately, the apico-basal polarity is lost, and cytoskeletons are reorganized to define cell shape and adapt for cell motility [10]. Cortical actin cytoskeleton undergoes dynamic rearrangement to form sheet-like lamellipodia and spike-like filopodia during EMT, enabling cell elongation and directional migration [11]. In addition, microtubule cytoskeleton plays a key role in EMT program as well. For example, histone deacetylase 6 (HDAC6), an important microtubule-binding protein, is a crucial regulator of EMT by deacetylating α-tubulin [12, 13].

There are two types of cilia in lung tissues, motile cilia emerging from airway epithelial cells and primary cilia from alveolar epithelial cells. The former beat periodically to clear out harmful inhaled materials from the lung, whereas the latter are non-motile antenna-like protrusions, serving as highly conserved sensory organelles to perceive and integrate extracellular cues to regulate cellular activities [14–16]. Interestingly, TGF-β receptors and their downstream effectors SMAD2/3 and SMAD4, as well as several proteins from noncanonical TGF-β signaling pathways, localize to primary cilia [17–19]. Emerging evidence has implicated an intricate interplay between the EMT and primary ciliogenesis in physiological and pathological contexts [20–22]. However, whether and how this interplay regulates pulmonary fibrosis remains unclear. In this study, we sought to investigate the roles of EMT and primary ciliary homeostasis in pulmonary fibrosis, aiming to explore the targetable molecules for the treatment of pulmonary fibrosis.

## Materials and methods

### Reagents and chemicals

Bleomycin sulfate (BLM), chloral hydrate (CH), TGF-β pathway inhibitor SB431542 (SB), 4′,6-Diamidine-2′-phenylindole dihydrochloride (DAPI), prostaglandin E2 (PGE2), and antibodies against γ-tubulin (#T6557), acetylated α-tubulin (#T6793), HDAC6 (#SAB2106255), aphla-smooth muscle actin (#A2547), vimentin (#V2258), Cylindromatosis (CYLD, #SAB4200060), and β-actin (#66009-1-Ig) were purchased from Sigma-Aldrich. Antibodies against α-tubulin (#Ab18251), E-cadherin (BD Bioscience, #610182), N-cadherin (BD Bioscience, #610920), Arl13b (Proteintech, #17711-1-AP), and GAPDH (Proteintech, #10494-1-AP), as well as HRP-conjugated secondary antibodies (Solarbio, #SE131) were obtained from the indicated sources. TRITC- and FITC-conjugated secondary antibodies were purchased from Jackson ImmunoResearch Laboratories. Recombinant human transforming growth factor β1 (rhTGF-β1, hereafter referred as TGF-β) was purchased from Proteintech (#100-21).

### Cell culture and transfection

A549 cells were purchased from the American Type Culture Collection and were cultured in Dulbecco’s modified Eagle’s medium supplemented with 10% fetal bovine serum at 37 °C in 5% CO_2_. Primary mouse alveolar epithelial type II cells (pMATII) were obtained from Fenghui Biotechnology (Changsha, China) and cultured in specialized culture medium (Fenghui Biotechnology). Cells were tested for mycoplasma contamination. Cells were treated with TGF-β for the indicated time to induce EMT program. For primary ciliogenesis, cells were starved for 24 or 48 h to enhance primary cilium formation [23, 24]. The sequence of siRNAs targeting CYLD and luciferase as a control were described previously [25]. siRNAs were transfected into cells using Lipofectamine RNAiMAX (Invitrogen) according to the manufacturer’s recommendations. For evaluation of the effect of EMT on primary ciliogenesis, cells at 50% confluency were treated with TGF-β (10 ng/mL) together with or without SB inhibitors for 48 h, followed by serum starvation for 48 h. For evaluation of the effect of primary cilia on EMT, cells at 50% confluency were serum-starved for 48 h, followed by TGF-β treatment (10 ng/mL) for 48 h.

### Wound healing assays

Wound healing assays were performed as described previously [26, 27]. In brief, confluent cells in 24-well plates were serum-starved overnight, and a scratch gap was created using a 10-μL pipette tip. Cells were then washed twice with phosphate-buffered saline (PBS) to remove the cell debris. Images of the scratch were captured at the indicated times, and the extent of wound closure was analyzed using Image-pro Plus software.

### Immunofluorescence

Cells were fixed with 4% paraformaldehyde (PFA) for 10 min, permeabilized with 0.1% Triton X-100 for 15 min, and blocked with 2% bovine serum albumin (BSA) for 30 min. Fixed cells were then probed in sequence with the indicated primary and secondary antibodies for 45-60 min. Nuclei were counterstained with DAPI. Coverslips were mounted with 90% glycerol and visualized with a Leica TCS SP8 confocal microscope. The percentage of ciliated cells was counted, and the length of cilia was measured with the Image J software. Only cilia with distinguishable ends were included for the measurement of the percentage of ciliated cells and the average ciliary length.

### Immunoblotting

Cell lysates were diluted in SDS loading buffer and incubated at 95 °C for 10 min. Proteins were resolved by SDS-PAGE and then transferred to polyvinylidenedifluoride (PVDF) membranes (Millipore). The membranes were blocked with 5% fat-free milk, followed by sequential incubation at room temperature with the indicated primary antibodies and horseradish peroxidase-conjugated secondary antibodies. Bound antibodies were detected using enhanced chemiluminescent detection reagent (Millipore). The densitometry of immunoblot bands were quantified with the Image J software. The first lane of each immunoblot was taken as control and set up to be 1.0, and the other lanes were normalized to the first lane.

### BLM-induced pulmonary fibrosis

Male C57BL/6 mice, *hdac6*^−/−^ or *cyld*^−/−^ (8 weeks old) were intratracheally injected with single dose of BLM (2.5 U/kg) or an equal volume of saline to induce pulmonary fibrosis, each group contains three mice and no randomization was used. Briefly, the mice were anesthetized with 7.5% chloral hydrate solution, and a 1 cm incision in the neck area of the mice was made using a surgical blade, followed by bluntly dissected and exposed the trachea. After intratracheal injection of BLM using a syringe, the skin at the incision site was clamped, and the wound was sutured using wound clips. Mice were then held upright and gently tapped on the back to ensure even distribution of the solution in the lung. Mice were sacrificed at day 14 and the lung tissues were isolated for further study. The use and operation of mice were in accordance with relevant regulations and approved by the Animal Care and Use Committee of Nankai University.

### Masson’s trichrome staining

Masson’s trichrome staining is used for semi-quantitative analysis of collagen fiber deposition in pulmonary fibrosis tissue. with blue-stained collagen fibers indicating positive expression. Lung tissues were fixed, embedded, sectioned, and stained using Masson’s trichrome staining reagent (Solabao, Beijing, China) according to the manufacturer’s manual. The sections were visualized with a light microscope.

### Statistical analysis

All experiments were repeated independently a minimum of three times. Data are blinded analyzed by an independent person and expressed as mean ± standard error of the mean (SEM) without applying any exclusion criteria. Significant differences were assessed using Student’s *t*-test for pairwise comparisons and analysis of variance (ANOVA) for comparison of multiple groups. *P*-values less than 0.05 were considered statistically significant.

## Results

### Primary cilia are suppressed in bleomycin (BLM)-induced pulmonary fibrosis

**Fig. 1:**
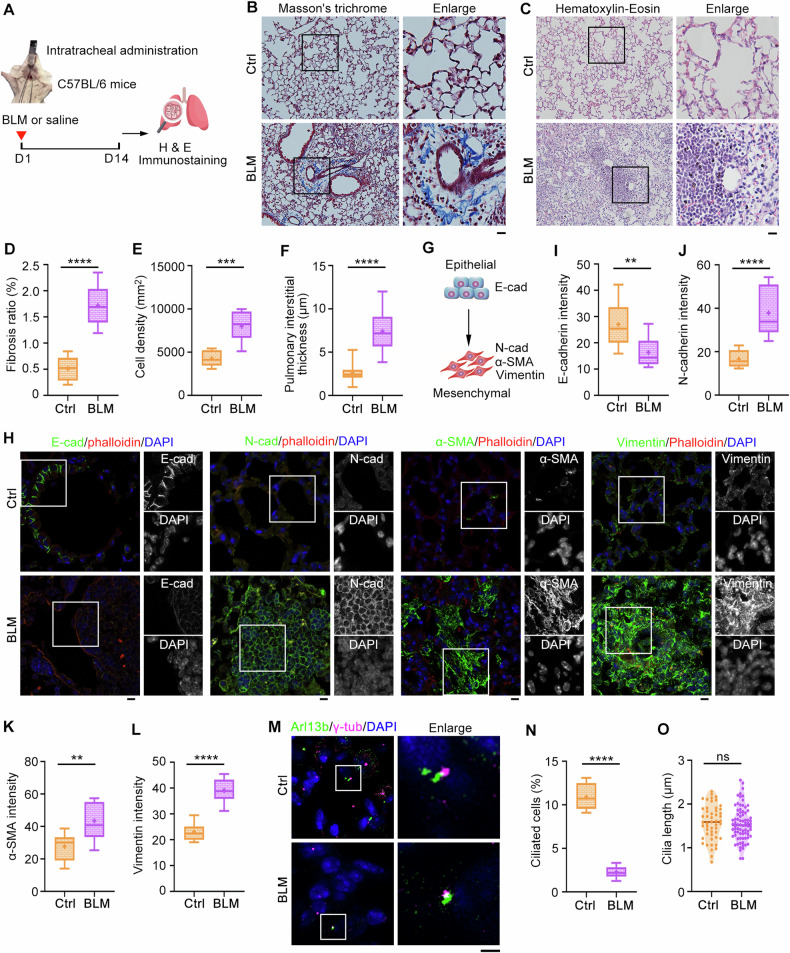
A close correlation between EMT and primary cilia during pulmonary fibrosis. **A** An illustration depicting bleomycin (BLM)-induced pulmonary fibrosis mouse model. Lung tissue sections from saline- or BLM-treated mice were subjected to Masson′s trichrome (**B**) and hematoxylin-eosin (H & E) staining (**C**), and fibrosis ratio (**D**), cell density (**E**), and pulmonary interstitial thickness (**F**) were quantified accordingly. *n* = 3 mice in each group. Scale bars, 5 μm. **G** An illustration showing the changes of marker proteins during EMT. Lung tissue sections from saline- or BLM-treated mice were immunostained with the indicated antibodies (**H**), and fluorescence intensities of E-cadherin (**I**), N-cadherin (**J**), α-SMA (**K**), and vimentin (**L**) were quantified. *n* = 3 mice in each group. Scale bars, 5 μm. Lung tissue sections from saline- or BLM-treated mice were immunostained with the indicated antibodies (**M**), and the percentage of ciliated cells (**N**) and ciliary length (**O**) were quantified. *n* > 50 cells. Scale bars, 5 μm. **P < 0.01; ***P < 0.001; ****P < 0.0001; ns not significant.

To investigate whether the interplay between EMT and primary cilia is critically involved in the pathogenesis of pulmonary fibrosis, we used the BLM-induced mouse model to recapitulate pulmonary fibrosis. C57BL/6 mice were intratracheally administrated with bleomycin or 0.9% NaCl and lung tissues were isolated at day 14 (Fig. 1A). Masson′s trichrome and hematoxylin-eosin staining revealed that the fibrosis foci was successfully formed in BLM-treated mice (Fig. 1B–F). Given that alveolar epithelial cells undergo EMT when damaged and play a critical role in the pathogenesis of pulmonary fibrosis, we examined this process by immunostaining with epithelial and mesenchymal-associated markers (Fig. 1G). As shown in Fig. 1H–L & Fig. S1, a significant decrease in E-cadherin and an increase in N-cadherin, α-smooth muscle actin (α-SMA), and vimentin were observed in BLM-induced pulmonary fibrosis group, confirming the activation of the EMT program. Here actin filaments were stained with phalloidin to show the cell morphology. Next, we immunostained with Arl13b (a ciliary marker) and γ-tubulin (a centrosome marker) for ciliary examination. Interestingly, BLM treatment resulted in a substantial decrease in the percentage of ciliated cells (Fig. 1M, N), although the ciliary length was almost not affected (Fig. 1M, O). Given that multiple signaling pathways are converged in EMT program and primary cilia, an interconnection between these two cell-biological programs may be the underlying cause of pulmonary fibrosis.

### EMT program attenuates ciliation in vitro

**Fig. 2:**
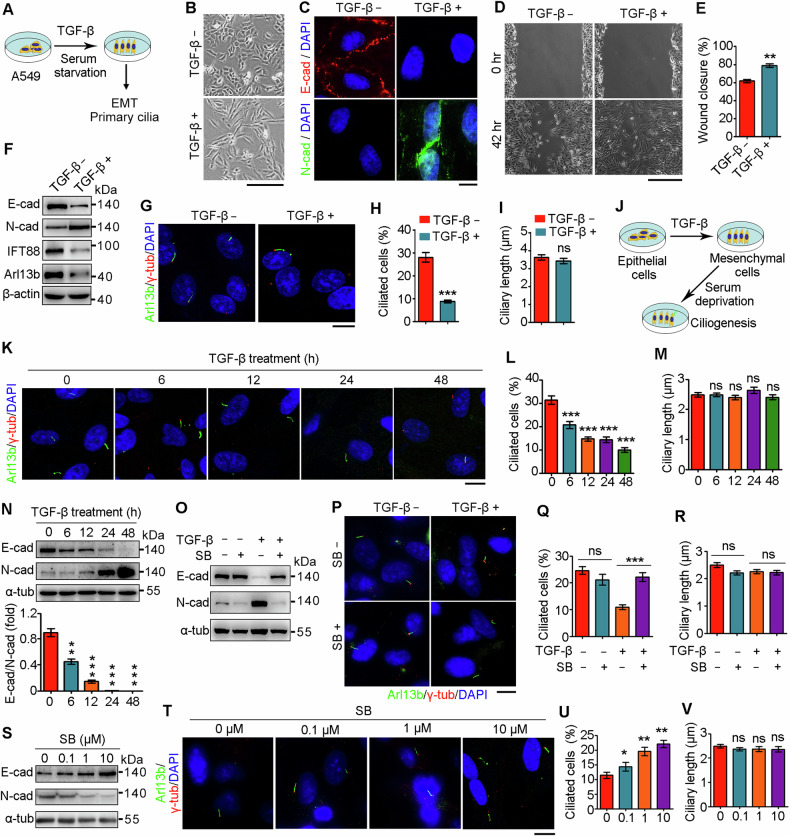
EMT regulates primary ciliogenesis. **A** A diagram showing the strategy to induce EMT and primary ciliogenesis by TGF-β exposure and serum starvation. (**B**) Morphology of A549 cells treated with or without TGF-β for 48 h. Scale bar, 100 μm. (**C**) Immunofluorescence images of A549 cells treated with or without TGF-β, followed by immunostaining with the indicated antibodies and DAPI. Scale bar, 5 μm. Wound healing images of A549 cells treated with or without TGF-β (**D**), and the wound closure was quantified (**E**). *n* = 3. Scale bar, 100 μm. A549 cells were treated with or without TGF-β, followed by immunoblotting (**F**) and immunostaining (**G**) with the indicated antibodies, and the percentages of ciliated cells (**H**) and ciliary lengths (**I**) were quantified. *n* > 50 cells. Scale bar, 5 μm. **J** A diagram showing the strategy to induce EMT and subsequent primary ciliogenesis. A549 cells were treated with TGF-β for the indicated time, followed by serum starvation for 48 h and immunostaining (**K**) and immunoblotting (**N**, *n* = 3) with the indicated antibodies, and the percentages of ciliated cells (**L**, *n* > 50 cells) and ciliary lengths (**M**, *n* > 50 cells) were quantified. Scale bar, 5 μm. **O**–**R** A549 cells were treated with or without TGF-β and SB431542 for 48 h, followed by serum starvation for 48 h and immunoblotting (**O**) and immunostaining (**P**) with the indicated antibodies, and the percentages of ciliated cells (**Q**) and ciliary lengths (**R**) were quantified. Scale bar, 5 μm. A549 cells were treated with TGF-β, together with various concentrations of SB431542 for 48 h, followed by serum starvation for 48 h and immunoblotting (**S**) and immunostaining (**T**) with the indicated antibodies, and the percentages of ciliated cells (**U**) and ciliary lengths (**V**) were quantified. *n* > 50 cells. Scale bar, 5 μm. **P < 0.01; ***P < 0.001; ns not significant.

Given that alveolar epithelial cells play a critical role in the pathogenesis of pulmonary fibrosis, we challenged primary mouse alveolar epithelial type II cells (pMATII) and A549 cells (human lung alveolar epithelial type II cells) with TGF-β in serum-free medium to induce EMT and primary cilium formation (Fig. 2A). TGF-β exposure transformed the morphology of pMATII and A549 cells from a polygonal, cobblestone phenotype into a spindle-shaped, mesenchymal phenotype (Fig. 2B and Fig. S2A), indicative of the occurrence of EMT. Immunofluorescence microscopy and immunoblot analysis revealed that TGF-β treatment repressed the expression of E-cadherin while increasing the expression of N-cadherin (Fig. 2C and Fig. S2A), confirming successful induction of the EMT by TGF-β. Consistent with acquisition of a mesenchymal phenotype, wound healing assays revealed that TGF-β treatment promoted cellular motility (Fig. 2D, E). Importantly, accompanied by TGF-β-induced EMT, the expression of IFT88 and Arl13b, two crucial proteins for ciliogenesis [28, 29], was attenuated (Fig. 2F and Fig. S2B). Consistently, the percentage of ciliated cells was significantly decreased upon TGF-β treatment, although the length of the primary cilia was not affected (Fig. 2G–I and Fig. S2B–D).

To examine the causality of EMT program and primary cilia, we challenged A549 cells with TGF-β to induce the EMT program and subsequently starved in serum-free medium to induce primary cilium formation (Fig. 2J). Time-course analysis revealed that the percentage of ciliated cells gradually decreased as the duration of TGF-β application increased (Fig. 2K, L), although increased duration had little effect on ciliary length (Fig. 2K, M). TGF-β exposure also resulted in altered expression of E-cadherin and N-cadherin in a time-dependent manner (Fig. 2N), indicating an essential role for the EMT program in primary ciliogenesis. We next blocked the TGF-β signaling with SB431542 to validate the specific effect of EMT program on primary ciliogenesis. As expected, SB431542 treatment abrogated TGF-β-induced EMT program (Fig. 2O), and promoted the formation of primary cilia without affecting their length (Fig. 2P–R). Moreover, SB431542 treatment abolished TGF-β-induced EMT program and ameliorated TGF-β-induced ciliary defects in a dose-dependent manner (Fig. 2S–V). Collectively, these findings suggest an intricate crosstalk between EMT and primary cilia and a critical role for the EMT program in primary ciliogenesis.

### CYLD is a critical regulator of EMT and ciliation

**Fig. 3:**
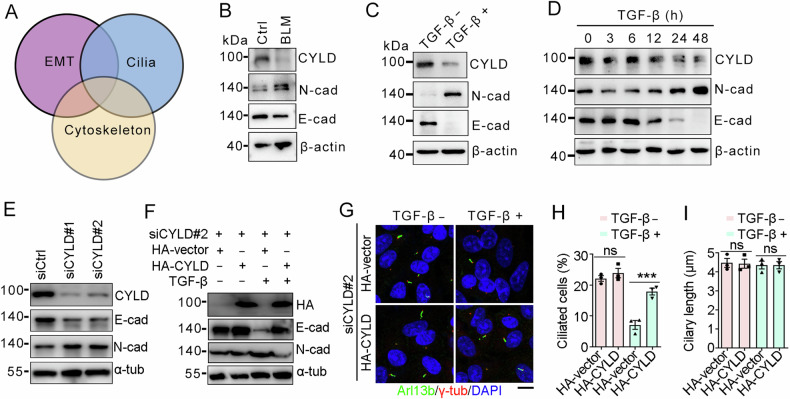
CYLD is a critical regulator for EMT and primary ciliogenesis. **A** A diagram showing the relationship between EMT, primary cilia, and cytoskeleton. Immunoblots of lung tissues (**B**) and TGF-β-treated A549 cells (**C**). **D** Immunoblots of A549 cells treated with TGF-β for the indicated time point. **E** Immunoblots of A549 cells transfected with siRNAs targeting CYLD. A549 cells were treated as indicated, followed by immunoblotting (**F**) and immunostaining (**G**) with the indicated antibodies, and the percentages of ciliated cells (**H**) and ciliary lengths (**I**) were quantified. *n* > 50 cells. Scale bar, 5 μm. ***P < 0.001; ns not significant.

To get insights into the molecular mechanism governing EMT program and primary cilia, we analyzed the regulator for these events. Given the critical involvement of cytoskeletal remodeling in both the EMT program and primary ciliogenesis [30–32], we focus on cytoskeleton-associated proteins (Fig. 3A). Interestingly, we found that CYLD, a microtubule-associated deubiquitinase, was remarkably downregulated in the BLM-treat lung tissues (Fig. 3B). Consistently, TGF-β treatment also decreased the expression level of CYLD (Fig. 3C), and this decrease corresponded to the changes of EMT markers in a time-dependent manner (Fig. 3D). In addition, depletion of CYLD promoted EMT program as demonstrated that E-cadherin was reduced while N-cadherin was increased (Fig. 3E). Reintroduction of CYLD could rescue TGF-β-induced EMT program (Fig. 3F), suggesting the specific role for CYLD in regulating EMT. Importantly, CYLD reintroduction was able to restore ciliary defects caused by TGF-β-induced EMT (Fig. 3G–I). These data indicate that CYLD is a critical regulator for EMT program and primary ciliogenesis.

### CYLD regulates EMT and ciliary homeostasis during pulmonary fibrosis

**Fig. 4:**
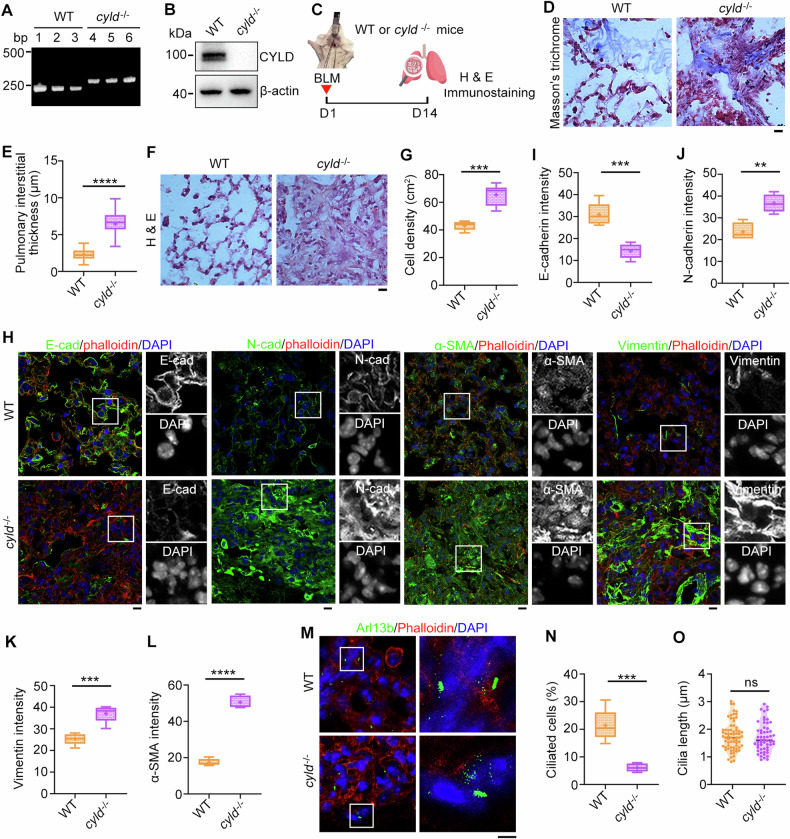
CYLD depletion aggravates pulmonary fibrosis. **A**, **B** PCR and immunoblotting analysis to confirm the knockout of *cyld* in mice. **C** An illustration depicting wildtype (WT) and *cyld*^−/−^ knockout mice treated with BLM to induce pulmonary fibrosis. Lung tissue sections from WT or *cyld*^−/−^ knockout mice were subjected to Masson′s trichrome (**D**) and H & E staining (**F**), and pulmonary interstitial thickness (**E**) and cell density (**G**) were quantified accordingly. *n* = 3 mice in each group. Scale bars, 5 μm. Lung tissue sections from WT or *cyld*^−/−^ knockout mice were immunostained with the indicated antibodies (**H**), and fluorescence intensities of E-cadherin (**I**), N-cadherin (**J**), vimentin (**K**), and α-SMA (**L**) were quantified. *n* = 3 mice in each group. Scale bars, 5 μm. Lung tissue sections from WT or *cyld*^−/−^ knockout mice were immunostained with the indicated antibodies (**M**), and the percentage of ciliated cells (**N**) and ciliary length (**O**) were quantified. *n* > 50 cells. Scale bars, 5 μm. **P < 0.01; ***P < 0.001; ****P < 0.0001; ns not significant.

Having identified the role of CYLD in EMT program and primary cilia, we next generated CYLD knockout mice to examine its influence on pulmonary fibrosis. PCR and immunoblotting analysis demonstrated that *cyld* gene was successfully deleted from the genome (Fig. 4A, B). Strikingly, CYLD deficiency significantly exacerbated BLM-induced pulmonary fibrosis as demonstrated that the pulmonary interstitial thickness and cell density was enhanced in the *cyld*^*−/−*^ knockout mice (Fig. 4C–G). Immunostaining of EMT markers revealed that deletion of CYLD led to a remarkable decrease in E-cadherin but an increase in N-cadherin, α-SMA, and vimentin, suggestive of the occurrence of EMT program (Fig. 4H–L and Fig. S3). Correspondingly, the percentage of ciliated cells was suppressed although the ciliary length was not affected (Fig. 4M–O). These data indicate that CYLD may be a critical regulator for EMT program and primary ciliary homeostasis during pulmonary fibrosis.

### HDAC6 is inactivated during pulmonary fibrosis

**Fig. 5:**
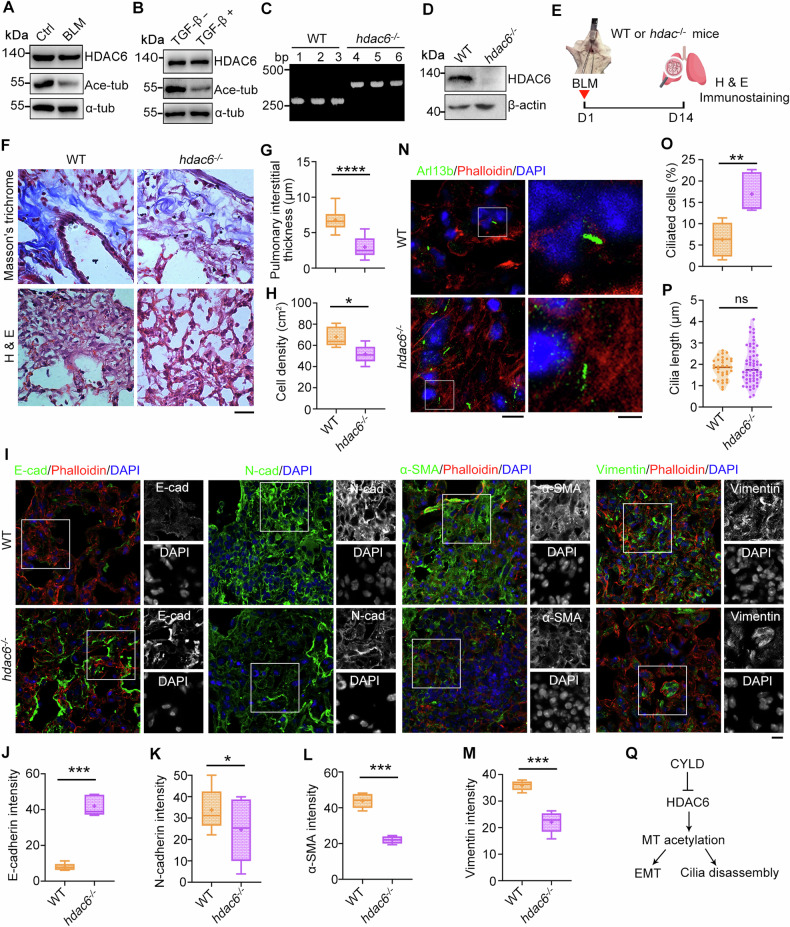
HDAC6 deficiency protects mice from BLM-induced pulmonary fibrosis. Immunoblots of lung tissues (**A**) and TGF-β-treated A549 cells (**B**) with the indicated antibodies. **C**, **D** PCR and immunoblotting analysis to confirm the knockout of *hdac6* in mice. **E** An illustration depicting wildtype (WT) and *hdac6*^−/−^ knockout mice treated with BLM to induce pulmonary fibrosis. Lung tissue sections from WT or *hdac6*^−/−^ knockout mice were subjected to Masson′s trichrome and H & E staining (**F**), and pulmonary interstitial thickness (**G**) and cell density (**H**) were quantified accordingly. *n* = 3 mice in each group. Scale bars, 5 μm. Lung tissue sections from WT or *hdac6*^−/−^ knockout mice were immunostained with the indicated antibodies (**I**), and fluorescence intensities of E-cadherin (**J**), N-cadherin (**K**), α-SMA (**L**), and vimentin (**M**) were quantified. *n* = 3 mice in each group. Scale bars, 5 μm. Lung tissue sections from WT or *hdac6*^−/−^ knockout mice were immunostained with the indicated antibodies (**N**), and the percentage of ciliated cells (**O**) and ciliary length (**P**) were quantified. *n* > 50 cells. Scale bars, 5 μm. **Q** A diagram showing the pathway underlying CYLD-mediated EMT and ciliary homeostasis. *P < 0.05; **P < 0.01; ***P < 0.001; ****P < 0.0001; ns not significant.

HDAC6 as a major tubulin deacetylase plays a crucial role in EMT program and ciliary homeostasis [33]. Thus, we examined whether HDAC6 is involved in pulmonary fibrosis. Although the level of HDAC6 was not affected in BLM-treated lung tissues, its deacetylase activity was enhanced as demonstrated that acetylated tubulin was reduced in both BLM and TGF-β treatment group (Fig. 5A, B). We next examined the role of HDAC6 in pulmonary fibrosis using BLM-treated *hdac6*^*−/−*^ knockout mice (Fig. 5C–E). Masson′s trichrome and hematoxylin-eosin staining demonstrated that HDAC6 deficiency protected mice from BLM-induced pulmonary fibrosis (Fig. 5F–H). Moreover, immunostaining of EMT markers revealed that HDAC6 deficiency promoted EMT program (Fig. 5I–M and Fig. S4). Simultaneously, the percentage of ciliated cells was elevated in lung tissues of BLM-treated *hdac6*^*−/−*^ knockout mice, although the ciliary length was not influenced (Fig. 5N–P). Our previous study and others revealed that CYLD is a HDAC6 inactivator [23, 34], we speculate that CYLD regulates EMT program and ciliary homeostasis by inactivating HDAC6 during pulmonary fibrosis (Fig. 5Q).

### Manipulation of primary ciliary homeostasis is an efficient means to regulate EMT program

**Fig. 6:**
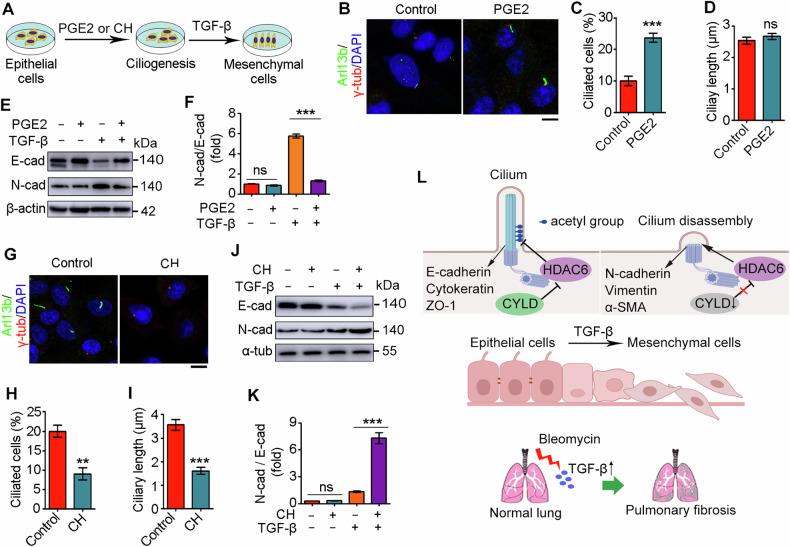
Primary cilia play an important role in regulating EMT. **A** A diagram showing the strategy to investigate the role of primary cilia in EMT. Immunofluorescence images (**B**), percentage of ciliated cells (**C**), and ciliary length (**D**) of A549 cells treated with PGE2 for 48 h, followed by TGF-β treatment for 48 h. Scale bar: 5 μm. A549 cells were treated with PGE2 for 48 h, followed by TGF-β treatment for 48 h and immunoblotting with the indicated antibodies (**E**), and the intensities of N-cadherin/E-cadherin were quantified (**F**, n = 3). Immunofluorescence images (**G**), percentage of ciliated cells (**H**), and ciliary length (**I**) of A549 cells treated with chloral hydrate (CH) for 48 h, followed by TGF-β treatment for 48 h. *n* > 50 cells. Scale bar: 5 μm. A549 cells were treated with CH for 48 h, followed by TGF-β treatment for 48 h and immunoblotting with the indicated antibodies (**J**), and the intensities of N-cadherin/E-cadherin were quantified (**K**, n = 3). **L** A proposed model showing CYLD/HDAC6 signaling-mediated interplay between EMT and primary ciliary homeostasis during pulmonary fibrosis. **P < 0.01; ***P < 0.001; ns not significant.

Regarding the critical role for primary cilia in various signaling pathways, we next investigated whether primary cilia could modulate EMT program. We experimentally altered primary ciliary homeostasis by treating cells with ciliary modulators prior to TGF-β treatment (Fig. 6A). PGE2, an eicosanoid that facilitates intraflagellar transport [35], promoted the formation of primary cilia, although it did not affect the length of primary cilia (Fig. 6B–D). In the absence of TGF-β, PGE2 pretreatment had little effect on the expression of E-cadherin and N-cadherin (Fig. 6E, F). By contrast, in the presence of TGF-β, PGE2 pretreatment markedly reduced the ratio of N-cadherin to E-cadherin (Fig. 6E, F), indicating that ciliated cells are more resistant to undergo the EMT program. Next, we treated A549 cells with chloral hydrate to induce primary cilium disassembly (Fig. 6G–I). Chloral hydrate pretreatment had no effect on the EMT program in the absence of TGF-β, however, it remarkably promoted the ratio of N-cadherin to E-cadherin upon TGF-β activation, confirming the suppressive role for primary cilia in the EMT program (Fig. 6J, K). Collectively, these data suggest that targeting primary cilia may be a potential strategy for the treatment of pulmonary fibrosis.

## Discussion

Pulmonary epithelium is susceptible to environmental exposures, such as cigarette smoke, inhalation of dust, infection, and gastroesophageal reflux. Chronic or repeated injury of epithelium leads to ectopic expression of profibrotic mediators, such as TGF-β, which induce EMT program and the differentiation of fibroblasts into myofibroblasts and the formation of fibrotic foci. Primary cilia in lung alveolar epithelial cells serves as an important sensory organelle that perceives and converts environmental cues into intracellular signals [36]. However, whether and how the interconnections between the EMT and primary cilia affect pulmonary fibrosis remains largely unknown. In this study, we identify a critical role for CYLD/HDAC6 signaling in regulating the reciprocal interplay between the EMT program and primary cilia during pulmonary fibrosis. In the normal conditions, CYLD inactivates HDAC6 to promote primary ciliary homeostasis as well as inhibit EMT program; however, during pulmonary fibrosis, CYLD deficiency relieves its inhibitory interaction with HDAC6, thereby leading to primary disassembly and the EMT program **(**Fig. 6L).

Our in vitro and in vivo data revealed that EMT program is associated with primary cilium defects, indicating the mesenchymal cells with enhanced motility are prone to be non-ciliated. In line with our findings, Rozycki et al. reported that epithelial–myofibroblast transition (EMyT) contributes to the loss of primary cilia [37]. Regarding the existence of partial EMT, temporally seamless tracing of EMT status is warranted for the accurate examination of the association between EMT and ciliary homeostasis during pulmonary fibrosis [38]. Importantly, we found that promoting or removing primary cilia was able to reverse or accelerate EMT program, suggesting that targeting primary cilia is a promising strategy for the treatment of pulmonary fibrosis. However, it is noted that the role of primary cilia in EMT program might be cell-type dependent, as primary cilia are essential for EMT-associated program in basal mammary tumor-initiating cells to acquire stemness properties, but seem to be dispensable for maintenance of the mesenchymal-like phenotype [39, 40]. In addition, both the percentage of ciliated cells and ciliary length are attenuated during EMT of kidney cells [40]. However, in the present study, we demonstrated that EMT program restrained primary cilium formation without affecting ciliary length, supporting the cell-type dependent mechanism in these processes.

Acetylated α-tubulin, which represents stabilized microtubules, is an important regulator of EMT and primary ciliary homeostasis [41, 42]. HDAC6 as the major deacetylase of α-tubulin plays a critical role in both EMT and primary ciliary homeostasis. In this study, we found that the acetylated α-tubulin was significantly reduced but the level of HDAC6 was not changed during pulmonary fibrosis. In support of our findings, Gu et al. revealed that TGF-β treatment leads to HDAC6 activation without influencing its expression level. Moreover, we found that CYLD is downregulated during pulmonary fibrosis, and *cyld*^*−/−*^ knockout mice were susceptible to BLM-induced pulmonary fibrosis, suggesting the significance of CYLD in lung function. Consistently, Lim et al. observed the protective role for CYLD in streptococcus pneumoniae infection-induced pulmonary fibrosis [43]. Since CYLD is a microtubule-binding protein as well as a deubiquitinase, it is not surprising that CYLD elicits its action via different signaling pathways. Interestingly, we found that *hdac6*^*−/−*^ knockout mice were resistant to BLM-induced pulmonary fibrosis. Considering the inhibitory interaction of CYLD with HDAC6, we speculate that CYLD regulates EMT and primary ciliary homeostasis by inactivating HDAC6. Given that CYLD as a deubiquitinase and HDAC6 as a deacetylase have a number of substrates, it is tempting to investigate other molecular mechanisms underlying CYLD/HDAC6-mediated pulmonary fibrosis and the therapeutic potential for treating this terrible disease with inhibitors targeting CYLD and HDAC6.

### Supplementary information

Supplementary Figures

Supplementary material

## Data Availability

Research data supporting the findings of this study are available within the paper in the main text or the Supplementary file. Full and uncropped western blots are included in the Supplementary Material.
