# Supplementary material for: CYLD/HDAC6 signaling regulates the interplay between epithelial-mesenchymal transition and ciliary homeostasis during pulmonary fibrosis
Source: Cell Death Dis. 2024 Aug 9;15(8):581. doi: 10.1038/s41419-024-06972-4 (PMC11316090; doi:10.1038/s41419-024-06972-4)
Supplement: Supplementary file 1 — Supplementary Figures [file 41419_2024_6972_MOESM1_ESM.docx]

**Supplementary information**

**CYLD/HDAC6 signaling regulates the interplay between epithelial-mesenchymal transition and ciliary homeostasis during pulmonary fibrosis**

Hua Ni^1,2,#^, Miao Chen^3,#^, Dan Dong^1,#^, Yunqiang Zhou^1^, Yu Cao^4^, Ruixin Ge^4^, Xiangrui Luo^4^, Yutao Wang^2^, Xifeng Dong^5^, Jun Zhou^1,4^, Dengwen Li^1,*^, Songbo Xie^6,*^, Min Liu^7,*^

^1^ State Key Laboratory of Medicinal Chemical Biology, Haihe Laboratory of Cell Ecosystem, College of Life Sciences, Nankai University, Tianjin 300071, China

^2^ Key Laboratory of Biological Resources and Ecology of Pamirs Plateau in Xinjiang Uygur Autonomous Region, College of Life and Geographic Sciences, Kashi University, Kashi 844000, China

^3^ School of Life Sciences and Medicine, Shandong University of Technology, Zibo 255500, China

^4^ Center for Cell Structure and Function, Collaborative Innovation Center of Cell Biology in Universities of Shandong, College of Life Sciences, Shandong Normal University, Jinan 250014, China.

^5^ Department of Hematology, Tianjin Key Laboratory of Bone Marrow Failure and Malignant Hemopoietic Clone Control, Tianjin Institute of Hematology, Tianjin Medical University General Hospital, Tianjin 300052, China.

^6^ Department of Ophthalmology, Tianjin Medical University General Hospital, Ministry of Education International Joint Laboratory of Ocular Diseases, Tianjin Key Laboratory of Ocular Trauma, Tianjin Institute of Eye Health and Eye Diseases, China-UK "Belt and Road" Ophthalmology Joint Laboratory, Haihe Laboratory of Cell Ecosystem, Tianjin Medical University, Tianjin 300052, China.

^7^ Laboratory of Tissue Homeostasis, Haihe Laboratory of Cell Ecosystem, Tianjin 300462, China

^#^These authors contributed equally to this work.

***Correspondence authors**

E-mail addresses: dwli@nankai.edu.cn (D.L.); songboxie@tmu.edu.cn (S.X.); minliu@nankai.edu.cn (M.L.).

**Running title:** CYLD mediates EMT and ciliary homeostasis

**Supplementary Figures**


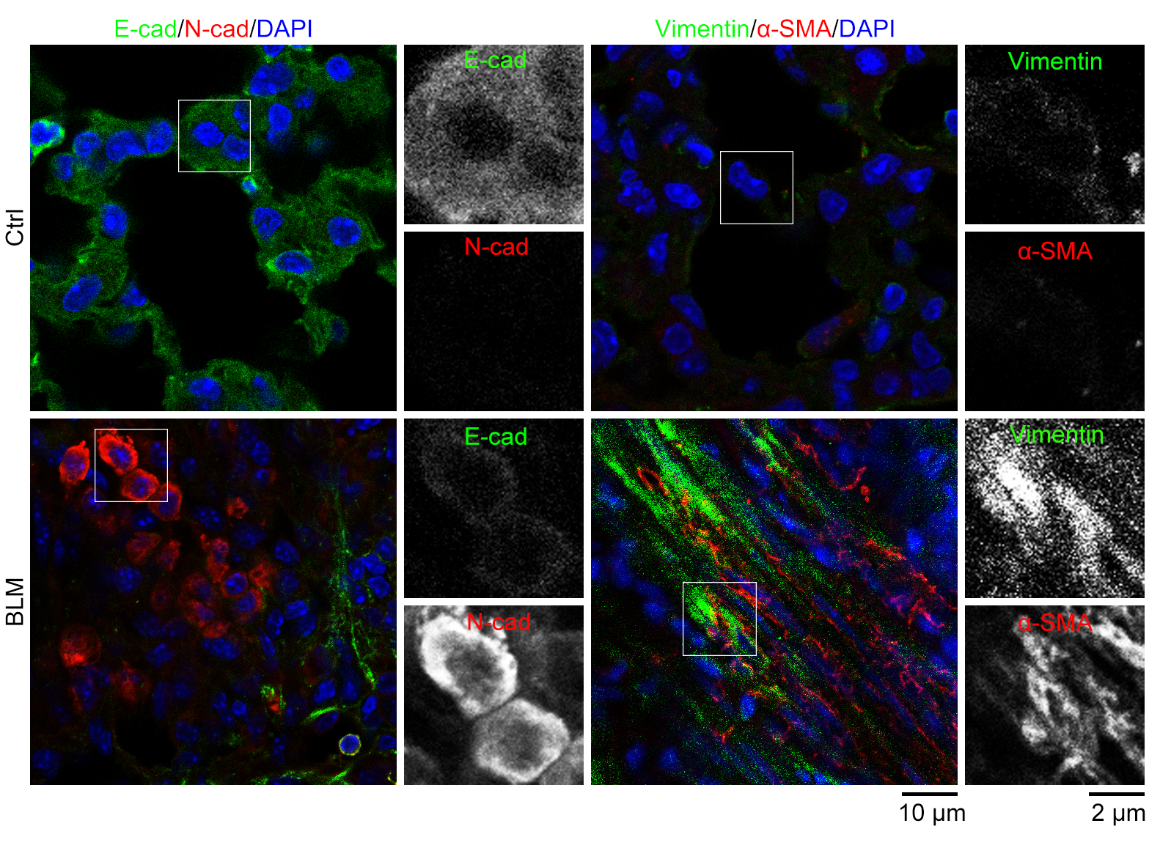


**Figure S1.** Lung tissue sections from saline- or BLM-treated mice were immunostained with the indicated antibodies and DAPI.


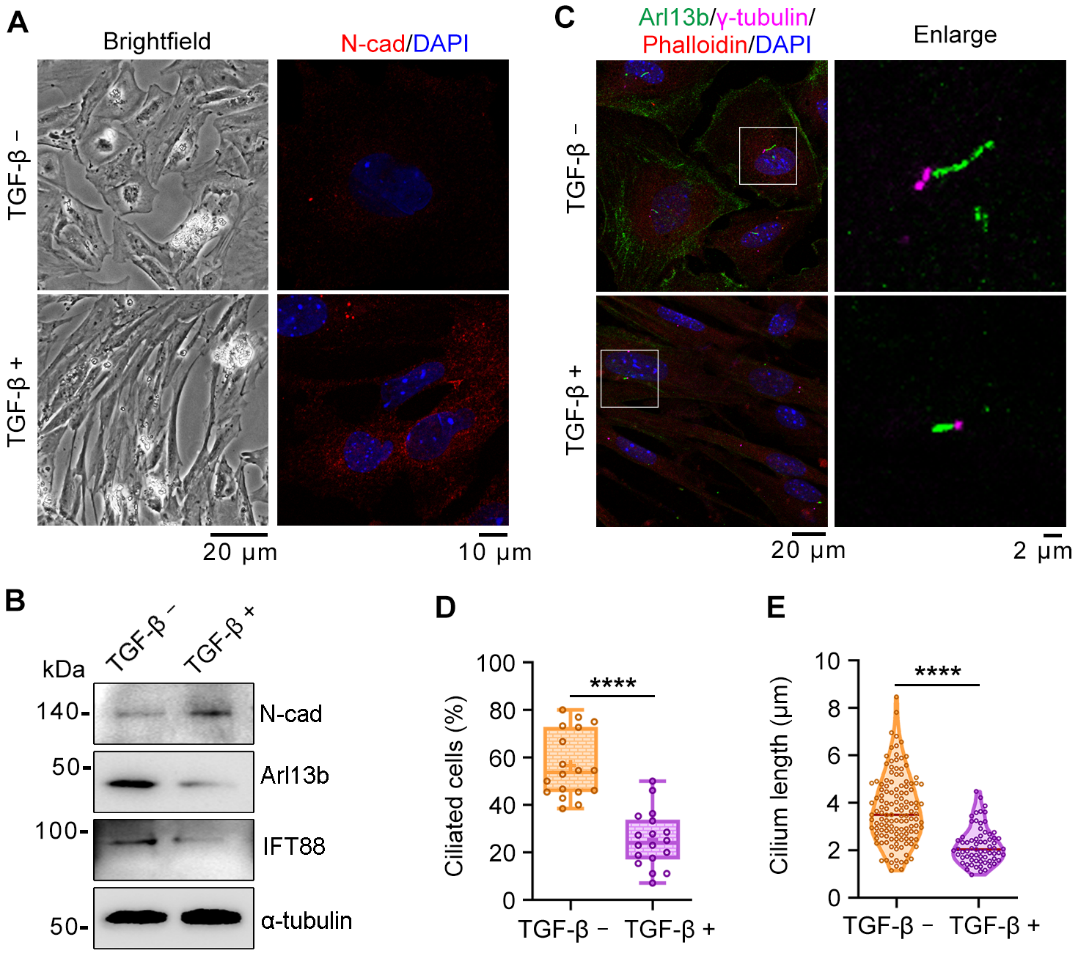


**Figure S2. An association between EMT and cilium formation.** (A) Morphology and immunofluorescence images of primary mouse alveolar type II cells treated with or without TGF-β for 48 hours. (B) Immunoblots of primary mouse alveolar type II cells treated with or without TGF-β. (C-E) Immunofluorescence images of primary mouse alveolar type II cells treated with or without TGF-β for 48 hours, followed by immunostaining with the indicated antibodies and DAPI (C). The percentages of ciliated cells (D, n > 50 cells from 3 independent experiment) and ciliary lengths (E, n >100 cells from 3 independent experiment) were quantified.


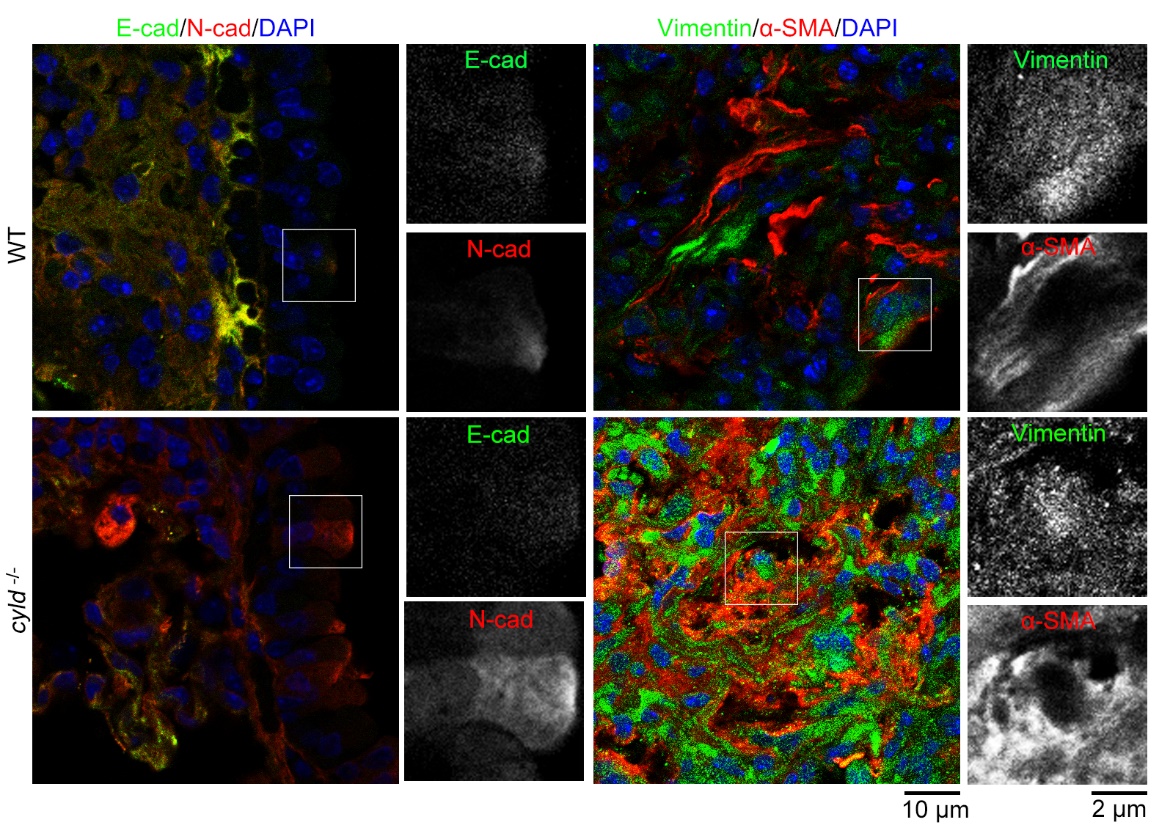


**Figure S3.** Lung tissue sections from WT or *cyld^-/-^* knockout mice were immunostained with the indicated antibodies and DAPI.


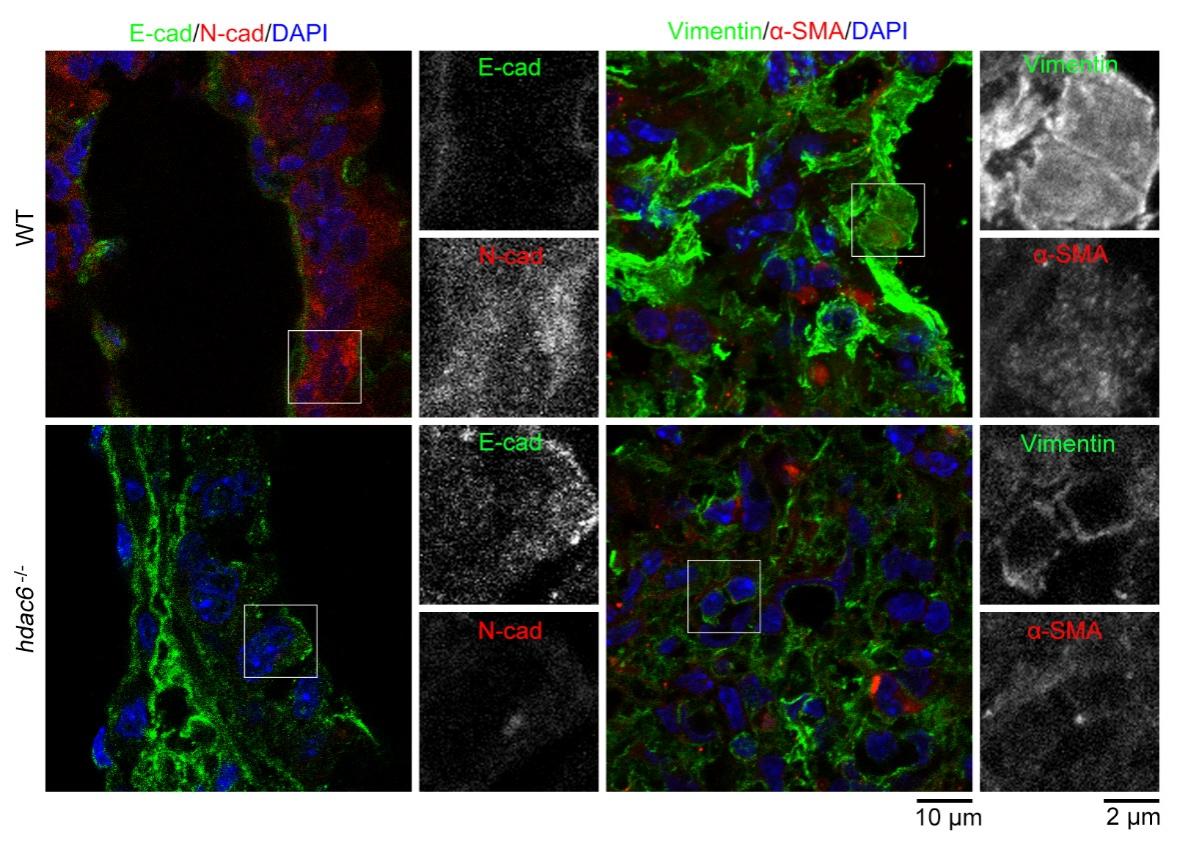


**Figure S4.** Lung tissue sections from WT or *hdac6^-/-^* knockout mice were immunostained with the indicated antibodies and DAPI.
