# Supplementary figures and images for: CYLD/HDAC6 signaling regulates the interplay between epithelial-mesenchymal transition and ciliary homeostasis during pulmonary fibrosis
Source: Cell Death Dis. 2024 Aug 9;15(8):581. doi: 10.1038/s41419-024-06972-4 (PMC11316090; doi:10.1038/s41419-024-06972-4)

Fig 2F

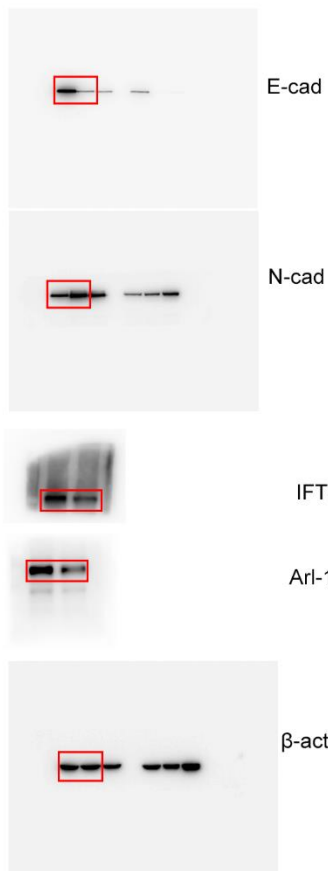

Fig 2N

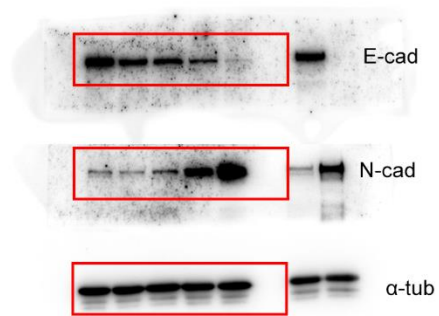

Fig 2O

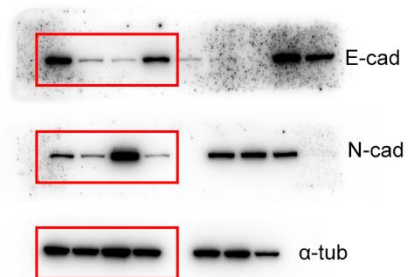

Fig 2S

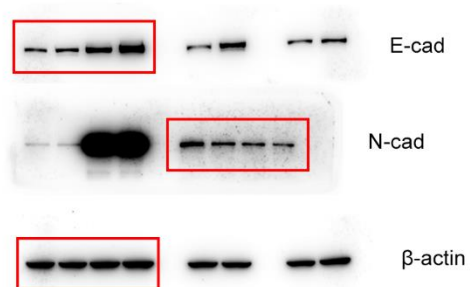

Fig 3B

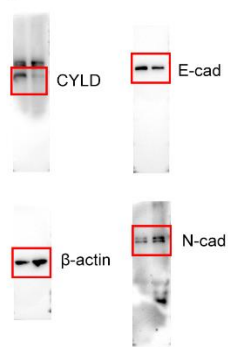

Fig 3C

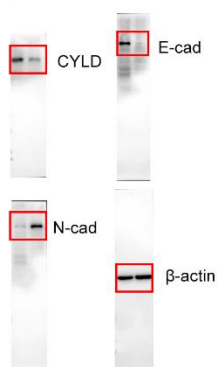

Fig 3D

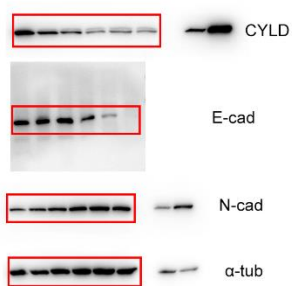

Fig 3E

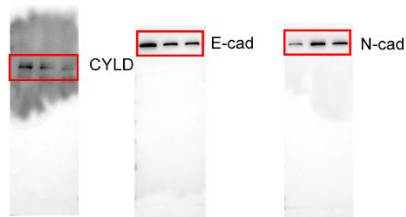

Fig 3F

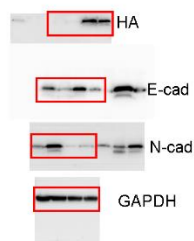

Fig 4B

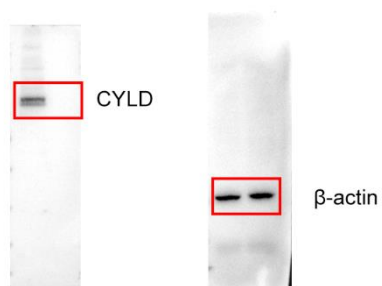

Fig 5A

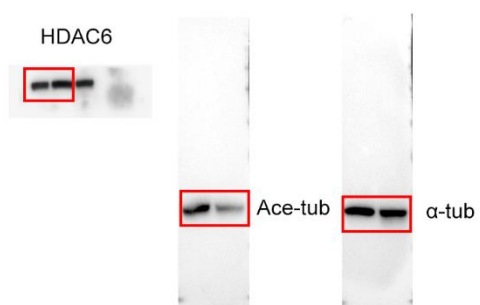

Fig 5B

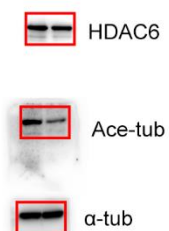

Fig 5D

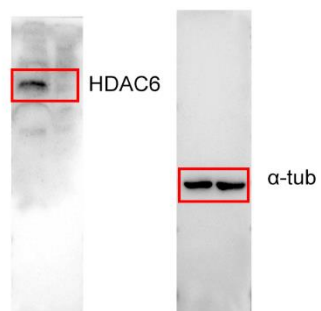

Fig 6E

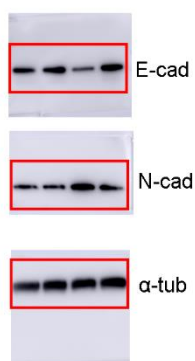

Fig 6J

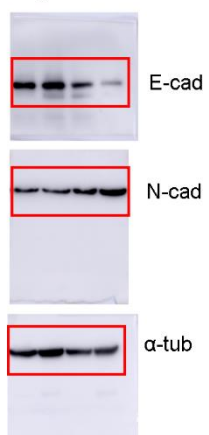

Fig.S2B

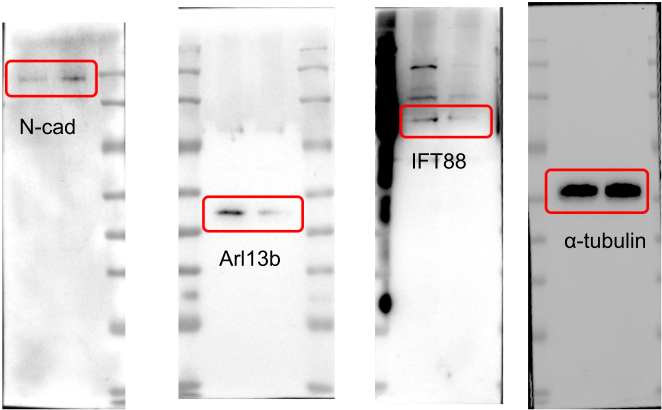

Supplement: Supplementary file 2 — Supplementary material [file 41419_2024_6972_MOESM2_ESM.pdf]
